# Use of Anterior Lumbar Interbody Fusion as a Revision Approach Following Failed Posterior Instrumented Lumbar Fusion: A Case Report

**DOI:** 10.7759/cureus.107828

**Published:** 2026-04-27

**Authors:** Eduardo Córdova Fuentes, Joab D Olivo Gómez, Luis Garibay Infante, José C Sauri Barraza, Eduardo Callejas Ponce

**Affiliations:** 1 Spine Surgery, Centro Médico ABC, Santa Fe, MEX; 2 Neurosciences, Hospital Zambrano Hellion, Nuevo Leon, MEX; 3 Spine Surgery, Clínica de Especialistas en Columna Vertebral, Centro Médico ABC, Ciudad de México, MEX

**Keywords:** alif, indirect decompression, interbody fusion, lumbar spine, lumboradiculopathy

## Abstract

A suitable option for revision surgery for posterior approach that permits avoiding fibrosis is the anterior lumbar interbody fusion (ALIF), which is also used to treat painful degenerative disc disease and restore the sagittal balance of the spine.

A 64-year-old female patient presented with a 20-year history of lumboradiculopathy. She had undergone a treatment of therapeutic facet and foraminal block of L4-L5, L5-S1, left laminoforaminotomy L4-L5, right L5-S1 and posterior lumbar interbody fusion L4-L5 and L5-S1. During the procedure, a fistula was identified in the L5-S1 space. Therefore, it was decided to maintain the level without interbody fusion.

She came to our medical unit three months after the last instrumentation, complaining of pain in the lumbar region with radiation to the left leg, which is disabling and limits her activities of daily living. Cabinet studies showed posterior instrumentation from L4 to S1, lateral interbody cage at L4-L5, L4-L5, Meyerding grade I spondylolisthesis with instability in the sagittal plane of L5-S1, and lateral recess stenosis left L5-S1.

An L5-S1 anterior lumbar interbody fusion was performed and bars and screws from S1 were removed via a posterior approach. This led to a radiological improvement by achieving an adequate sagittal balance. Patient's symptoms and pain were reduced by indirectly performing decompression of the spinal nerve. The procedure did not have any complications.

This case emphasizes the importance of presurgical planning in multi-treated patients with previous fusions without improvement and the choice of an anterior lumbar interbody fusion, which shows greater safety, clinical and radiological improvement for patients.

## Introduction

Lumboradiculopathy secondary to degenerative lumbar spine disease is a common cause of disability, particularly in patients with a history of prior spinal surgery. In this population, persistent or recurrent symptoms are frequently associated with pseudarthrosis, residual or recurrent foraminal stenosis, segmental instability, or implant failure. Revision lumbar spine surgery is required in up to 10%-20% of cases, depending on the underlying pathology and surgical technique [1,2].

Revision procedures through a posterior approach are technically demanding due to epidural fibrosis, perineural adhesions, and altered anatomical landmarks, which increase the risk of dural injury, neural damage, and intraoperative complications [3].

Anterior lumbar interbody fusion (ALIF) has emerged as an effective alternative in this setting, as it allows access to the intervertebral disc space while avoiding posterior scar tissue. From a biomechanical standpoint, the anterior approach enables placement of larger interbody implants, increasing the surface area for fusion and facilitating restoration of disc height and lumbar lordosis [4,5].

Posterior interbody fusion techniques, such as posterior lumbar interbody fusion (PLIF) and transforaminal lumbar interbody fusion (TLIF), have demonstrated high fusion rates and favorable clinical outcomes [6]. However, complications such as pseudarthrosis, cage migration, or persistent foraminal stenosis may occur in a minority of cases, particularly in revision settings or in patients with risk factors. Smoking is a well-established risk factor for pseudarthrosis due to its negative effects on bone healing [7]. In such high-risk patients, bone morphogenetic proteins (BMP) have been proposed to enhance fusion rates [8]. This, in turn, contributes to indirect neural decompression through foraminal re-expansion and improvement of sagittal alignment [9].

Despite these advantages, the specific role of ALIF as a salvage technique in patients with prior posterior instrumentation remains insufficiently characterized, particularly in complex clinical scenarios [10].

The aim of this study is to describe the use of ALIF as a rescue technique in a patient with persistent lumboradiculopathy following prior posterior lumbar instrumentation, highlighting its clinical and radiological implications.

## Case presentation

A 64-year-old female patient with a medical history significant for hypertension, hypothyroidism, and prior myocardial infarction treated with coronary stenting presented with a 20-year history of lumboradiculopathy. She had undergone multiple conservative and interventional treatments, including facet and foraminal blocks at L4-L5 and L5-S1, as well as left L4-L5 and right L5-S1 laminoforaminotomies, without significant clinical improvement.

**Figure 1:**
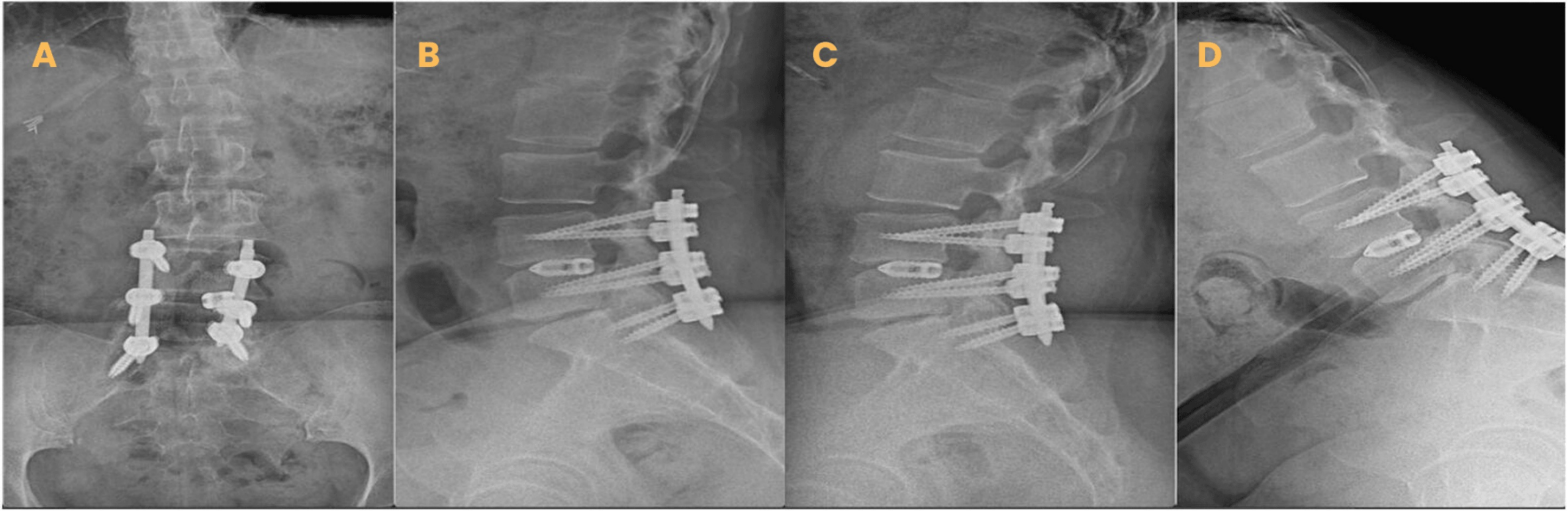
Radiography of lumbar spine. A, anteroposterior projection; B, lateral projection; C and D, dynamic projections. Images with posterior lumbar instrumentation L4-L5 + PLIF L4-L5

One year prior to presentation, she underwent posterior lumbar instrumentation from L4 to S1, combined with posterior lumbar interbody fusion (PLIF) at L4-L5 and L5-S1. During that procedure, a dural tear with cerebrospinal fluid (CSF) leak was identified at L5-S1; therefore, interbody fusion at this level was not performed (Figure 1).

Three months after surgery, the patient presented with persistent low back pain radiating to the left lower extremity, significantly limiting her daily activities. Physical examination revealed an antalgic gait, pain on lumbar extension, bilateral dysesthesia in L4-L5 dermatomes, preserved muscle strength (5/5), and a positive straight leg raise test on the left side.

**Figure 2:**
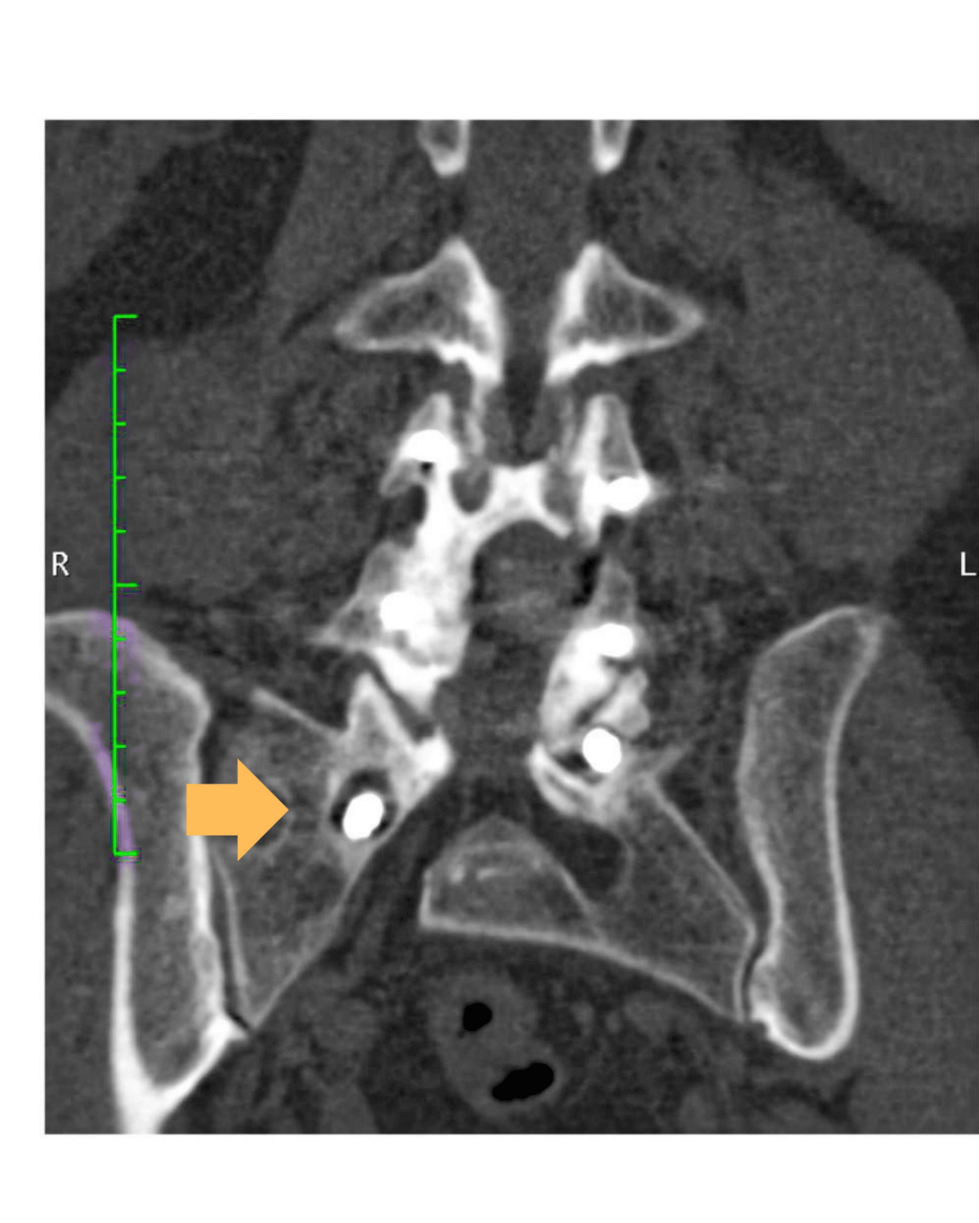
Computed tomography sagittal section of the lumbar spine with right L4–L5 facet fusion, with void sign at L5–S1

**Figure 3:**
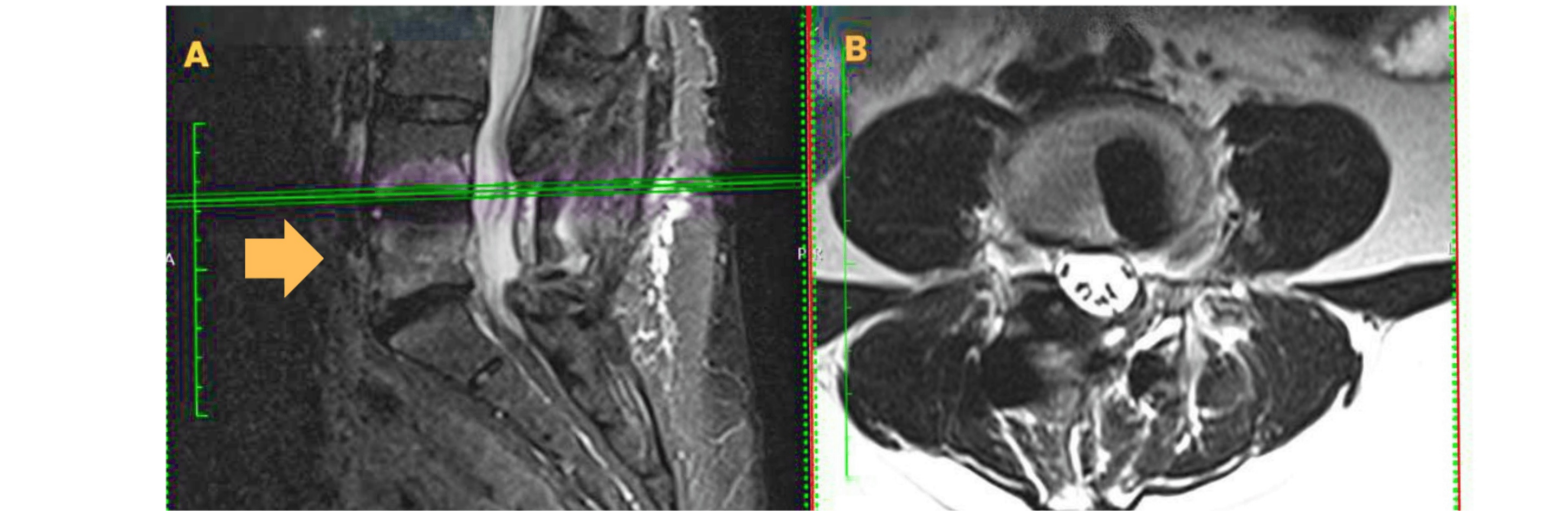
Magnetic resonance of the lumbar spine. A, coronal section; B, axial section. Images with left L4–L5 foraminal stenosis and bullet-shaped cage

Imaging studies demonstrated posterior instrumentation from L4 to S1 and a posterior interbody cage (PLIF) at L4-L5. There was no clear evidence of solid fusion in some segments. Additionally, grade I Meyerding spondylolisthesis at L5-S1 with sagittal instability and left foraminal stenosis at this level were identified. The S1 screws appeared to have limited length and suboptimal medialization, which may have contributed to distal construct instability (Figures 2, 3).

A diagnosis of left L5-S1 lumboradiculopathy without neurological deficit secondary to foraminal stenosis was established, associated with mechanical low back pain likely related to implant loosening at S1.

**Figure 4:**
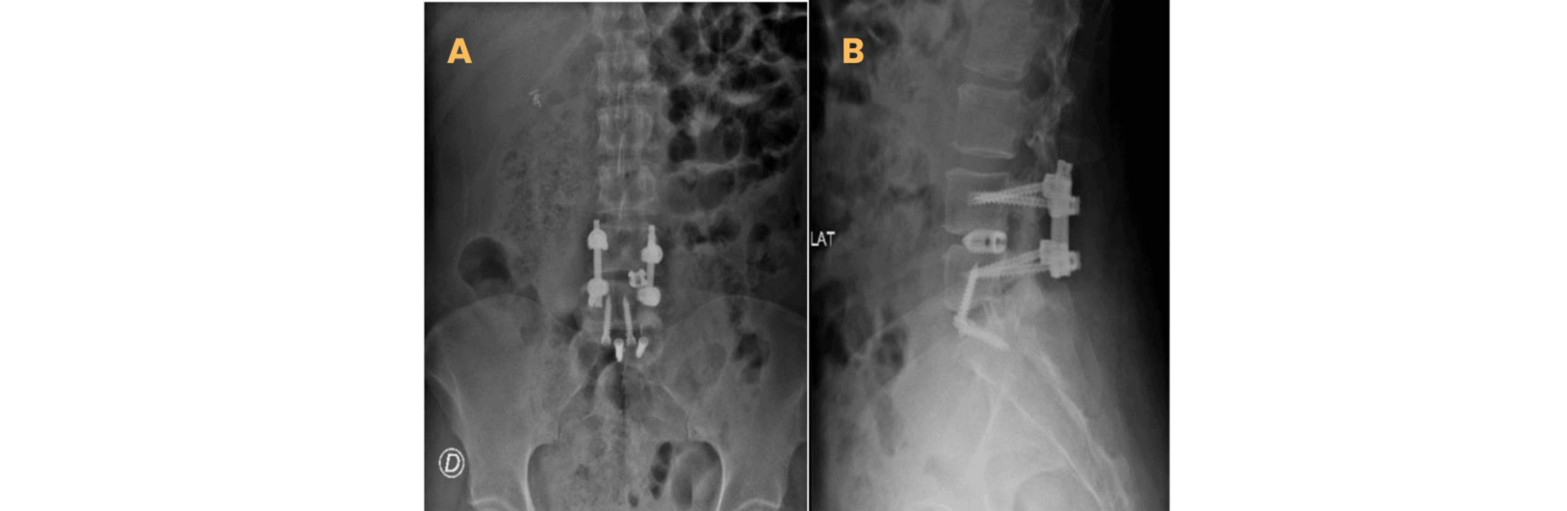
Radiography of lumbar spine. A, anteroposterior projection; B, lateral projection. Images with Anterior Lumbar Interbody Fusion (ALIF) L5–S1 + L3–L4 instrumentation + L3–L4 interbody cage

The patient underwent ALIF at L5-S1, combined with posterior removal of S1 screws and rods. Postoperatively, the patient experienced significant clinical improvement in pain. Radiological assessment showed qualitative improvement in sagittal alignment. No intraoperative or postoperative complications were observed (Figure 4).

## Discussion

Revision lumbar spine surgery remains a complex and challenging scenario, particularly in patients with prior posterior approaches. Epidural fibrosis and distorted anatomy increase the risk of complications and limit surgical exposure [3]. In this context, ALIF offers several advantages. The anterior approach allows direct access to the disc space without traversing scar tissue, reducing the risk of neural injury. It also facilitates placement of larger interbody implants, increasing the fusion surface area and enabling restoration of disc height and lumbar lordosis [4,5].

Restoration of disc height contributes to indirect decompression of neural elements through foraminal re-expansion, making ALIF particularly useful in cases of foraminal stenosis without severe neurological deficits [9]. Additionally, the anterior approach allows for more complete disc removal and release of the anterior longitudinal ligament, enhancing segmental and global alignment correction.

Posterior techniques such as PLIF and TLIF are well-established and effective [6]; however, their use in revision settings may be limited by technical challenges associated with scar tissue and previous instrumentation. ALIF has demonstrated fusion rates as high as 90%-97%, supporting its role in selected revision cases [5,10].

In the present case, the indication for ALIF was based on segmental instability, foraminal stenosis, and suspected implant failure at S1 in a previously instrumented spine. The anterior approach allowed effective treatment while avoiding reoperation through scarred posterior tissues, resulting in significant clinical improvement.

## Conclusions

ALIF is an effective surgical option as a rescue technique in patients with persistent lumboradiculopathy following prior posterior lumbar instrumentation. Its ability to avoid scar tissue, achieve indirect decompression, and restore spinal biomechanics makes it a valuable alternative in carefully selected revision cases.
